# The Impact of Household Debt on the Health of the Elderly in China: Evidence from China Family Panel Studies

**DOI:** 10.3390/ijerph20042946

**Published:** 2023-02-08

**Authors:** Ziyu Liu, Ke Zhao, Jinquan Liu, Yongfu Liu

**Affiliations:** School of Economics and Statistics, Guangzhou University, Guangzhou 510006, China

**Keywords:** health level, population aging, household debt, IV-Oprobit model

## Abstract

With the aging of China’s population and the expansion of household debt, the health of the elderly has become an important social issue. Based on the 2018 China Family Panel Studies (CFPS) database, we explored the impact of household debt on the health of older adults and the mechanism of transmission. The Oprobit and IV-Oprobit models were employed for our analysis. Results: (1) Household debt had a significant negative impact on both the physical and mental health of older adults. (2) Female older adults were more sensitive to the impact of household debt. Additionally, a higher education level led to an increasing impact of debt on mental health, but physical health was only affected in the low-education group. (3) The impact of household debt had an inverted U-shape relationship with household income, indicating that, as household income increases, the impact on health level first rises and then reduces after peaking at a middling level of income. (4) According to the mechanism analysis, household debt affects the health of the elderly by causing them to return to work and reducing their medical expenditures. Considering the above conclusions, we put forward some policy implications to alleviate the health problems of the elderly.

## 1. Introduction

Population aging has become one of the most prominent social problems in the world, including China. The proportion of elderly people in China is gradually rising; the proportion of the resident population aged 65 and over increased from 6.9 percent in 1999 to 14.2 percent in 2021, which is approximately 200.56 million people [1]. Additionally, the health problems of the elderly have become more serious. According to the “Report on the Development of Quality of Life for the Elderly in China (2019)”, approximately one-fourth of the elderly rated their health as poor, and stated that it became worse when they got older [2]. Older adults are not only facing physical health problems, such as chronic diseases, but also psychological factors such as loneliness, depression, and anxiety, which can deteriorate the mental health of the elderly. Considering the growing number and the social cost of the elderly, the health of older residents is not only related to themselves, but also to the medical caretaking burden and the supply and demand of labor from society. Therefore, the health of the elderly has become an important research field.

Meanwhile, with the continuous reform and opening, China’s economic growth and individual income growth have both maintained a high rate. However, household debt has also accumulated sharply, with the household leverage (the ratio of household debt to the annual GDP) in China having increased from 18.2 percent in 2008 to 65.1 percent in 2020 [3]. Household debt is an important socioeconomic determinant of individual health [4]. It can increase individual health by smoothing income and promoting consumption, but it can also reduce health by the corresponding financial stress [5]. Due to the lack of solvency, conservative consumption attitudes, and greater health risks, older adults are more vulnerable to the effect of household debt. With the aging of the population and the expansion of household debt, the impact of household debt on the health of the elderly and its mechanism of action have attracted the attention of scholars.

Previous studies on the impact of household debt on the health of the elderly were not consistent. First, there are different points of view on the impact of household debt on health. According to lifecycle theory, some studies find that access to credit is effective in alleviating households’ credit constraints and increasing health expenditures, with a positive impact on health [6]. However, others find that household debt puts financial stress on family members, forcing them to reduce medical spending and inducing psychological stress, anxiety, and depression [7,8], which have a negative impact on the health of the elderly [9]. Second, some studies found a strong statistical correlation between household debt and severe psychological problems [10,11]. However, most studies on this phenomenon have taken place in developed countries. In the UK, credit card debt had a significant positive correlation with depression [12]. Using data from 8400 respondents to four national surveys in the United States between 1994 and 2008, Sweet et al. found that financial indebtedness was significantly associated with worsening psychological stress, depression, and self-rated health [13]. Zurlo et al. found that in the US, the amount of debt and the occurrence of debt were significant predictors of depressive symptoms and lower psychological wellbeing [14]. Although there have been a few studies focusing on the effect of household debt on health in Asia [15], the results are still undetermined. Third, household debt may have different effects on different populations. Some studies found that household debt has a negative impact on middle-aged and older adults, but has a positive impact on younger adults [16]. Household debt is more damaging to the health of older people than younger people, with older debtors more likely to exhibit severe symptoms of depression, anxiety, and anger, in large part due to their fear of being unable to repay their debts [9]. Moreover, older adults tend to associate debt with shame, which can further exacerbate the psychological burden of debt [17]. Therefore, considering the inherent vulnerability of the elderly, it is important to explore the impact of household debt on the health of the elderly in China.

Besides the different impacts of household debt, there are different views about the transmission mechanisms. The first potential mechanism is returning to work. Older adults are usually retired from their work. However, an agreement has not yet been reached regarding the relationship between retirement and health. On one hand, retirement may improve health because it frees people from the burdens of work so that they can enjoy life [18,19,20]. Eibich found that retirement significantly improves the physical and mental health of older adults [21]. On the other hand, retirement may lead to changes in their range of social activities and habits and reduce their self-worth [22,23]. Additionally, because of the financial stress, older adults might return to work. Some studies have found a positive impact of post-retirement re-employment on the health level of older adults [24,25], but other studies have found that retired older adults sacrifice their health for work until their work capacity is lost due to higher household debt [26]. The second potential mechanism is lower medical expenditures. Heavy debt burdens can create financial strains that may force individuals to reduce their health expenditures [27]. Grossman found that consumers maximize their lifetime utility by making health investments and choices about the consumption of other goods under budget constraints. Hence, a decline in household income can reduce the household health budget [28,29,30,31]. As household debt increases, it leads to a decline in health due to the crowding out of other expenditures and lower medical expenditures [32]. Baneijee found that for US residents over the age of 50, medical expenditures accounted for the second largest share of total expenditures, and health levels decreased as health care spending decreased [33].

Most of the above literature focuses on the impact of household debt on health in developed countries such as Europe and the US, but few studies have focused on developing countries, especially China. There is also still a gap regarding the heterogeneity of factors such as gender, education level, and household income in the elderly population. Finally, the transmission mechanism studies were limited and produced inconclusive results. Therefore, based on the 2018 CFPS database, we evaluated the impact of household debt on the health levels of older adults in China, and solved endogeneity by employing an IV-Oprobit model. We analyzed the heterogeneity of the impact of household debt on different elderly groups, and examined the transmission mechanism.

Compared with previous studies, the contributions of this study are as follows: First, previous studies focused on the impact of household debt from the perspective of developed countries, and few studies have been conducted in China, so we have extended the research perspective. Second, in order to solve the endogeneity problem, we used the IV-Oprobit model and PSM to fill that gap. Third, based on gender, education level, and household income level, we identified the heterogeneity of the impact on different groups of older adults, in order to avoid structural problems. Finally, previous studies have focused on the impact of household debt, and there is still a lack of attention being paid to the transmission mechanisms, which is one of our contributions.

The remainder of this study is structured as follows: Section 2 describes the data and methodology. Section 3 represents the empirical results. Section 4 provides a discussion of the results. Section 5 presents our conclusions.

## 2. Materials and Methods

### 2.1. Data Source and Description

We used the China Family Panel Studies (CFPS) database as our data source. It is a national, multidisciplinary, social tracking survey project provided by the Institute of Social Science Survey (ISSS) of Peking University with a national representative sample of village (neighborhood), family, and family member follow-up surveys. The CFPS database contains over 16,000 households from 25 provinces, autonomous regions, and municipalities in China. The CFPS database reflects China’s family situation and economic activity, including the aspects of living conditions, family composition, and health status. Four waves of surveys have been conducted, in 2012, 2014, 2016, and 2018. Due to the increasing household debt, we only used the 2018 survey in this study. We mainly concentrated on the impact of household debt on the health level of older residents, aged over 60 years. A total of 7875 samples were selected after sample selection and data cleaning.

### 2.2. Variable Selection

#### 2.2.1. Explained Variables

Table 1 represents the variables and their definitions. The core explained variables in this study are the physical health and mental health of the elderly in China. According to Yang and Ma [34,35], the self-rated health and depression scores were selected as proxy variables, respectively. Self-rated health is a comprehensive assessment of a respondent’s health status that combines subjective experiences of both acute and chronic, and both fatal and non-fatal illnesses with their own disease and disability status, and fully represents an individual’s health. It is generally obtained by directly asking respondents relevant questions about their perceived health status, and is a more realistic reflection of residents’ health status because they know their own health status quite well. In this study, question P201 was selected as a variable. The question is “How do you think of your health?”, and the answers range from “poor” to “very good”, which are converted into ordered numbers 1–5. The higher the score, the better the respondent’s health. Furthermore, the mental health of older adults was measured using the depression score we constructed through the Center for Epidemiologic Studies of Depression (CES_D). The CFPS questionnaire contained six CES-D questions, including QN406, QN407, QN411, QN414, QN418, and QN420. Each question was scored from 1–4, and we added up the scores for the six questions. Additionally, the depression score indicated the degree of depressive symptoms experienced by the older adults surveyed; the higher score, the more depressed they are.

#### 2.2.2. Explanatory Variables

We chose household debt as the explanatory variable, where household debt refers to the sum of the housing loans and non-housing loans of the surveyed households in 2018. To prevent differences in magnitudes between data, we further logarithmized the debt data before estimation.

#### 2.2.3. Other Variables

Three types of control variables were selected for the estimation. First, demographic characteristic variables, including gender, age, education, urban resident status, and the presence of a drinking problem, were selected. Second, household control variables, including household income, household assets, and household size, were selected. Just like household debt, we logarithmized the household income and assets. Third, province control variables were selected.

### 2.3. Model Design

In order to analyze the impact of household debt on the physical health and mental health of older adults, we used the ordered probit (Oprobit) model to evaluate the impact. In this study, the model is constructed as follows:

(1) $$Health_{i}^{*}=\alpha_{1}+\beta_{1}Debt_{i}+\gamma_{1}X_{i}+v_{i}+\varepsilon_{i}$$

(2) $${\mathrm{Depression}}_{i}^{*}=\alpha_{2}+\beta_{2}Debt_{i}+\gamma_{2}X_{i}+v_{i}+\varepsilon_{i}$$

(3) $$Health_{i}=\{\begin{array}{ccc} 1 & if & \;\;\;\;\;Health_{i}^{*}\leq r_{1}\\ 2 & if & r_{1}<Health_{i}^{*}\leq r_{2}\\ 3 & if & r_{2}<Health_{i}^{*}\leq r_{3}\\ 4 & if & r_{3}<Health_{i}^{*}\leq r_{4}\\ 5 & if & r_{4}<Health_{i}^{*}\;\;\;\;\; \end{array}$$

(4) $$Depression_{i}=\{\begin{array}{ccc} 6 & if & \;\;\;\;\;\;Depression_{i}^{*}\leq s_{1}\\ 7 & if & s_{1}<Depression_{i}^{*}\leq s_{2}\\ 8 & if & s_{2}<Depression_{i}^{*}\leq s_{3}\\ \vdots & \vdots & \vdots \\ 24 & if & s_{18}<Depression_{i}^{*}\;\;\;\;\; \end{array}$$

where $Health_{i}^{*}$ represents self-rated health, $Depression_{i}^{*}$ represents the depression score, $Debt_{i}$ represents household debt, $X_{i}$ represents the control variables, including the demographic and household characteristics, $v_{i}$ represents the province fixed effect, and $\varepsilon_{i}$ represents the error term. The terms $r_{i}$ and $s_{i}$ represent the cut-off points, which are estimated below.

Furthermore, in order to solve the inverse causality, we used the instrumental variable ordered probit (IV-Oprobit) model to recalculate the original equations. According to Roodman [36], the IV-Oprobit model is constructed as follows:

(5) $$Debt_{i}=\delta_{1}Z_{i}+\theta_{1}X_{i}+u_{i}$$

(6) $$Health_{i}^{*}=\delta_{2}Debt_{i}+\theta_{2}X_{i}+v_{i}$$

where $Z_{i}$ represents the instrumental variable, and $u_{i}$ and $v_{i}$ represent the error terms of Equations (5) and (6), respectively. If there exists an endogeneity problem, the two error terms must satisfy $Cov\left(u_{i},v_{i}\right)\neq 0$.

Considering the self-selection bias, we used the propensity score matching (PSM) method to obtain a consistent value for the estimated average treatment effects on the treated (*ATT*). The calculation of the *ATT* is constructed as follows:

(7) $$ATT=E\left[Health_{1,i}|A_{i}=1\right]-E\left[Health_{0,i}|A_{i}=1\right]$$

where $Health_{1,i}$ represents the health of the elderly with household debt, and $Health_{0,i}$ represents the health of the elderly without household debt.

## 3. Results

### 3.1. Benchmark Empirical Analysis

Table 2 reports the results of the descriptive statistics. The mean value of self-rated health was 3.6889, indicating that most Chinese older adults in the sample were at a relatively healthy level. The depression score was 9.8987, which is lower than 15, indicating that the mental health of the elderly is also relatively good. The mean value of ln household debt was 2.4663. The average age of the elderly was 68.2160 years. Approximately half of respondents were male, and 47.02 percent of respondents lived in an urban area, indicating a reasonable distribution of the sample. The average education in years was 4.5363, indicating that the majority of the sample did not go to primary school. The mean value regarding the presence of a drinking habit was 17.51 percent, indicating that the proportion of alcohol drinkers was small. The mean value of ln household income was 10.2647 and the mean value of ln household assets was 12.5613, indicating that Chinese household assets were at a high level. The mean value of household size was 3.8031, indicating that the resident population of Chinese households was around four people, which is in line with China’s two-child policy. Before the empirical analysis, it was necessary to test whether there is potential collinearity. The results show that the mean value of the variance inflation factor (VIF) is 1.24, which is less than 10. Hence, there is no multicollinearity in our model.

Table 3 reports the empirical results of the Oprobit model on the impact of household debt on the physical and mental health of older adults in China. Columns 1 to 3 represent the empirical results for household debt on the physical health of older adults. Column 1 only shows the effect of the core explanatory variable and province control variable, which represents the simple relationship between household debt and the physical health of the elderly. Based on Column 1, Column 2 takes the demographic characteristics into consideration, and Column 3 further includes the household characteristics. Column 4 further replaced the province fixed effect with household fixed effect. It can be found from the results of Column 3 that the impact of household debt on the self-rated health of the elderly is −0.0072 and is significant at the 1% level. This indicates that the expansion of household debt has a significant negative impact on the physical health of older people. As household debt accumulates, the health of older adults deteriorates significantly. In addition, as can be seen from Columns 1 to 4, with more control variables and alternative fixed effect, the effect of household debt on health grows larger and more significant, indicating that our findings are stable.

Meanwhile, Columns 5 to 8 in Table 3 show the results of the impact of household debt on older adults’ mental health. The model is set up in exactly the same form as Columns 1 to 4. It can be seen in Column 6 that the impact of household debt on depression in the elderly is 0.0210, which means that household debt has a significant positive impact on depression score. This indicates that the expansion of household debt worsened the mental health of adults. Furthermore, from Columns 5 to 8, it can be seen that the coefficient of household debt remained significant for all results. Combining the above results, it can be seen that household debt has a significant negative impact on both the physical and mental health of the elderly.

Considering other control variables, some variables also affected the health level of older adults. At the individual level, higher age led to worse health, and women were more affected by household debt than men. The effect of educational attainment on self-rated health was not significant, but showed a significant positive effect on mental health. While alcohol consumption deteriorated the health of older adults, it also alleviated their psychological stress. At the household level, higher household income and assets were able to alleviate health problems and depression, but the impact of household size on the health of older adults was not significant.

Given that the coefficients of the Oprobit have no practical meaning, we further estimated the marginal effect. Table 4 reports the results of the marginal effects of household debt on the elderly’s health. Considering physical health, as household debt rises by one unit, the probability of the elderly having a poor health (health = 1) and average health (health = 2) status increased by 0.24% and 0.04%, respectively, and was significant at the 5% level. However, the probability of having good (health = 3), pretty good (health = 4), and very good (health = 5) health decreased by 0.10%, 0.07%, and 0.11%, respectively, and was significant at the 5% level. This indicates that the expansion of household debt would increase the probability of a bad health level and decrease the probability of a good health level. Similar to mental health, as household debt increased, the probability of a low depression level decreased, while the probability of a high depression level increased. Therefore, we can conclude that household debt has a negative impact on the physical and mental health of the elderly.

### 3.2. Endogeneity Analysis

The endogeneity in this study mainly results from omitted variables, reverse causality, and self-selection bias. First, we consider omitted variables bias. Taking many studies about the elderly’s health into consideration, we selected eligible variables to estimate our model. Furthermore, we applied the Ramsey’s RESET test to determine if there are omitted variables. The results show that the F values of the self-rated health and depression equations are 0.72 and 0.87, respectively, and the p values are 0.54 and 0.52, respectively; therefore, we cannot reject the hypothesis that the model has no omitted variables.

Second, we consider reverse causality. Household debt could lead to bad health, and vice versa. On one hand, higher household debt may reduce the household medical expenditure, leading to the poor health status of older people. On the other hand, poorer health can reduce labor hours and lower income levels, which can also raise household debt. In order to solve the endogeneity problem, we used the IV-Oprobit model for further analysis.

We selected household debt from the 2016 CFPS database as the instrumental variable. It was highly correlated with the explanatory variables, and was not correlated with the health of the elderly in 2018. We applied the correlation test to exam the correlation between the household debt in 2016 and the elderly’s health in 2018. The results showed that the coefficients of household debt in 2016 to self-rated health and depression of the elderly are 0.0093 and 0.0023, respectively, and neither are significant. Table 5 reports the empirical results of the IV-Oprobit model. First, Column 1 represents the first-stage estimation results. It shows that the correlation coefficient between household debt in 2018 and that in 2016 was 0.8977 and was significant at the 1% level. Meanwhile, the first-stage F-value was 1160.89, which rejected the hypothesis of weak instrumental variables, so it was appropriate to set the 2016 household debt as the instrumental variable. Second, the two-stage estimation and atanhrho_12 were both significant, indicating that the method effectively solves the endogeneity problem, and that the instrumental variable is appropriate. Finally, the results reveal that household debt has a significant negative effect on physical health and mental health, further confirming our findings in the benchmark empirical analysis.

Third, we consider self-selection bias. The propensity score matching (PSM) method can solve the problem of self-selection bias. In order to ensure the reliability of the results, we used the K-nearest-neighbor-matching method, Caliper matching method, and Kernel matching method to test our results. Table 6 reports the empirical results of PSM. In terms of physical health, the average ATT of three methods is −0.0810 and is significant at the 1% level, indicating that the self-rated health of the elderly with household debt is worse by 0.0810 than those without household debt. In terms of mental health, the average ATT of the three methods is 0.7253 and is significant at the 1% level, indicating that the level of depression of the elderly with household debt is 0.7253 higher than those without household debt. Although the magnitude of the impact of household debt on the health of the elderly differed, the symbols remained consistent with our benchmark empirical analysis. Therefore, the PSM results confirm our findings, suggesting that household debt has a significant negative impact on the physical and mental health of the elderly.

### 3.3. Robustness Test

After evaluating the impact of household debt on the health of the elderly and solving the problem of endogeneity, we tested the robustness of our findings through different indicators measured explanatory variables and different measurement methods.

In this study, we replaced household debt with house loans in 2018 and applied the Oprobit model to the health level of the elderly, while the corresponding control variables remained unchanged. In order to ensure the validity of the robust test, we excluded the top 20% and the bottom 20% of housing loan data. Table 7 reports the empirical results of the robustness test. Columns 1 and 2 reveal that, after replacing the variables, the impact remained negative and significant at the 1% level. Moreover, OLS was also implied as a measurement method in Columns 3 and 4. It can be seen that the impact of household debt on the health level of older people remained negative and significant at the 1% level. The above conclusions confirm the negative impact of household debt on the physical and mental health of older adults, indicating that our findings are robust.

### 3.4. Further Analysis

According to the results of benchmark model analysis, rising household debt impairs the health of the older population. However, the above conclusions have not explored the impact of household debt on different groups of older people, and the mechanism of impact of household debt on the health level of older adults remains ambiguous. Hence, in order to further understand the impact, we investigate the heterogeneity and transmission mechanism of household debt with regard to the health of older adults.

#### 3.4.1. Heterogeneity Analysis

In this section, we separate the older population based on their gender, education level, and household income level. We also estimated the marginal effects of each subgroup, and, due to the fluency, we reported the results in Appendix A.

Considering the different gender categories, the explained variables were separated into male older adults and female older adults. Table 8 reports the results regarding the heterogeneity of gender disparities. It can be seen that there exists heterogeneity between gender differences. First, Columns 1 and 2 reveal the estimation results of the effect of household debt on physical health for women and men, respectively. The impact on women was −0.00149 and was significant at the 1% level, but the impact on men was 0.0003 and insignificant. Second, Columns 3 and 4 demonstrate the results of the effect of household debt on the mental health of the two different groups. The coefficients were 0.0216 and 0.0205, respectively, and both were significant at the 1% level, indicating that the impact of household debt on female older adults is greater than that on male.

Moreover, Table A1 and Table A2 report the results of the marginal effects, respectively. The results showed that the marginal effects of household debt on the physical health were still not significant. Additionally, the probabilities of household debt on older female adults’ mental health were higher than that of the male counterpart, indicating that the marginal effect in the female subsample is larger than that of the male counterpart. In summary, after separating the whole sample by gender, the results show that household debt has a greater impact on the health of the female subsample than the male counterpart.

We further divided the whole sample into three subsamples based on years of education: a low level, including being illiterate; a medium level, including primary school and middle school; and a high level, including high school and beyond. Table 9 reports the results of heterogeneity of education disparities. Columns 1 to 3 show that the impact on older adults with a low education level was −0.0085 and was significant at the 5% level, but the impact on subsamples with a medium or high level of education was not significant. It can be seen that household debt had a negative impact on the physical health of the elderly with a low level of education. Meanwhile, Columns 4 to 6 show the impact of household debt on mental health at different education levels. The coefficients were 0.0171, 0.0215, and 0.0312, respectively, and all were significant at the 1% level.

Moreover, Table A3, Table A4 and Table A5 report the results of the marginal effects, respectively. Considering physical health, the marginal effects of household debt from the medium level and high level were not significant. However, in the low education level subgroup, it can be found that the probability of poor health rises and that of good health declines. Considering mental health, with the expansion of household debt, the highest probabilities of low depression were −0.35%, −0.61%, and −0.78% in each subgroup, which were significant at the 1% level. Additionally, the probabilities of high depression were 0.08%, 0.12%, and 0.19%, respectively, which were significant at the 1% level. Therefore, the results reveal that as the education level increases, household debt has a greater negative impact on the mental health of the elderly.

Finally, we divided the whole sample into three subsamples according to household income level. Those in the lower 25% of the sample in terms of income were set as the low-income group, the middle 50% were set as the middle-income group, and the upper 25% were set as the high-income group. In order to avoid the influence of extreme values, we removed the 1% tail data before estimation. Table 10 reports the results in terms of the heterogeneity of household income disparities. Columns 1 to 3 show that the impact of household debt on the physical health of the elderly was −0.0061, −0.0129, and −0.0047, respectively, and was significant at the 1% level. The results indicate that, as household income increased, the impact of household debt on physical health also increased, before falling after the middle-income level. Similarly, Columns 4 to 6 show that the negative impact of household debt on mental health peaked at the middle-income level and then fell, eventually forming an inverted U-shape curve.

Moreover, Table A6, Table A7 and Table A8 report the results of the marginal effects, respectively. Considering physical health, the highest probabilities of poor health were 0.21%, 0.49%, and 0.14%, respectively, which were all significant. In addition, the highest probabilities of good health were −0.10%, −0.18%, and −0.06%, respectively, which were all significant at 1% level. It can be found that the middle-income subgroup suffered the most, and the results of mental health showed the same conclusion. Therefore, the results show that the impact of household debt had an inverted U-shape relationship with household income.

#### 3.4.2. Mechanism Analysis

Through previous studies, we found that higher household debt leads to a decline in the physical and mental health of older adults, although an analysis of the mechanism is still needed. Existing research has found that there are two main mechanisms that affect the health of older adults. The first of these mechanisms is returning to work. With the increase in household debt, the elderly are forced back to work, or to extend their work time to pay off household debts; this extra working might worsen their health [37]. The second mechanism is a decrease in medical expenditure. Higher household debt leads to lower household disposable income, and household medical expenditures might be curtailed, thus worsening health [27]. Therefore, we selected “whether or not one worked in the past week” (yes = 1, no = 0) and personal health expenditure (logarithmized) as the mechanism variables to explore these two transmission mechanisms.

Table 11 reports the empirical results of the “returning to work” mechanism. Column 1 shows that household debt had a significant positive impact on the labor participation of the elderly. It is indicated that as household debt increases, the more likely it is that the elderly will have to rejoin work. Columns 2 and 3 show, after the inclusion of labor participation in the estimation, that labor had a significant negative impact on physical health and a significant positive impact on the depression score of the elderly, indicating that the labor participation of the elderly worsens their health. Meanwhile, household debt continued to have a significant negative impact on older adults’ health. Compared with Table 3, the coefficients of physical and mental health decreased from −0.0072 to −0.0070 and from 0.0210 to 0.0206, respectively, indicating that the impact of household debt was partially transferred to labor participation. Therefore, it can be seen that returning to work was an important mechanism of the effect of household debt on older adults’ health.

Table 12 reports the empirical results of the medical expenditure mechanism. Column 1 reveals that household debt had a significant negative impact on medical expenditure. This indicates that higher household debt reduced medical spending. Columns 2 and 3 reveal that the increase in medical expenditures improved the physical health of the elderly and relieved mental stress. Moreover, similar to the labor channel, the coefficients of household debt decreased compared with those in Table 3, indicating that the impact of household debt was partially transferred to medical expenditure. Therefore, it can be seen that medical expenditure is another important mechanism of the effect of household debt on the health of the elderly.

## 4. Discussion

We analyzed the impact of household debt on the physical and mental health of older adults based on 7578 samples. We constructed an Oprobit model to analyze the impact of household debt on the health of the elderly in China. We used IV-Oprobit to solve the endogeneity problem due to reverse causality, and solved the problems of omitted variables and self-selection bias. We also explored the heterogeneity and the transmission mechanism. The findings of this study have important practical implications for China as aging and household debt continue to rise.

We verified that household debt had a significant negative impact on the health of older adults in China. This is consistent with the findings of Gathergood [7]; it was found that household debt triggered mental stress and physical discomfort, especially when households had high repayment burdens. Additionally, the distress, anxiety, and stigma caused by the high burden further induced psychosomatic disorders and adversely affected individual health. Moreover, we also found that household debt has a negative impact on both the physical and mental health of older adults, which is consistent with the findings of Blazquez and Zurlo [14,16]. Using Spanish household data, the former found that household debt was associated with lower self-rated health among elderly individuals, while the latter, using US data, found that household debt also had a negative impact on mental health, due to depressive symptoms and decreased psychological wellbeing. Both studies showed that such an impact existed in developed countries, and we further verified that it also exists in developing countries, such as China. In addition, we also proved that our findings were robust by using alternative explanatory variables.

We found that older women are more stressed than the male counterparts by household debt, which is consistent with the results of Chen et al. [38]. It was found that women’s tendency to experience higher levels of stress for a given level of debt mediates the increased likelihood of women reporting that debt has affected their health. Meanwhile, we found that higher levels of education are associated with a larger impact of household debt on the mental health of elderly, and that household debt only affected physical health in the low-education group, which is not consistent with Sudore et al. [39]. This may be due to the fact that family health affects older adults through anxiety and depression as the level of education increases. Additionally, most of the low-education group were from a rural area, without knowledge and skills, and could only improve their pay by working harder. In such cases, their physical condition gradually deteriorates.

We found that with an increase in household income, the impact of household debt on the health of elderly represented an inverted U-shape relationship, i.e., the health of older adults from middle-income households was the most affected, which is inconsistent with the results of Amroussia et al. [40]. It was found that the health level of the elderly is positively correlated with income. The main difference between our findings may result from Chinese social customs and high house prices. Chinese people have a rigid demand for housing when it comes to aspects such as marriage and childbearing. In recent years, housing prices in China have been rising rapidly, and mortgage payments and related expenses have also increased. In response to financial pressures, the phenomenon of reverse repayment by older Chinese relatives to the next generation is also emerging significantly [41,42]. Whether this is a result of a decline in the total intergenerational resource allocation due to household debt, or the tendency of the parental generation to assume unlimited responsibility in intergenerational relationships [43], older family members view themselves as “debtors” with respect to the debts of their offspring. Therefore, the burden of household debt not only affects the head of the household and the adult offspring, but also inevitably affects the older members of the household. Middle-income households have to pay for daily expenses, child expenditure, and the health expenses of the elderly. Additionally, with the increase in housing prices, the need for parents to pay for the mortgage reduces the medical expenses of the elderly. Low-income families are mostly rural families who have self-built homes and are less affected by mortgages. The higher-income group is also less affected by debt than middle-income households because of their valuable assets.

By analyzing the transmission mechanism, we found that household debt affects the health level of the elderly through returning to work and a reduction in medical expenditure. First, due to the increasing pressure of household debt, older adults have to work longer or rejoin the workforce, which may impair physical health and induce psychological problems such as anxiety and depression, which is consistent with Benjamin et al. [37]. It was found that, due to poverty, older adults are not able to retire, and thus impair their health. Second, household debt reduces household income, thus forcing a reduction in health care expenditures and worsening the health problems of the elderly, which is consistent with Keese and Schmitz [27]. It was found that a heavy household debt burden raises the budget constraint and induces financial stress, which may force individuals to reduce their medical expenditures. The crowding-out effect of debt on health care consumption becomes more serious. On one hand, the reduction in disposable income forces households to cut back on expenditures and reduces spending on health. On the other hand, a high debt burden reduces the likelihood of older households being able to obtain credit, and households adopt a strategy of reducing expenditures to overcome future uncertainty, which leads to a negative impact on the health of the elderly.

## 5. Conclusions

According to the 2018 CFPS database, the Oprobit and IV-Oprobit model were used to measure the impact of household debt on the health of older adults, and the mechanisms driving its outcomes. The conclusions are as follows:

1. Household debt has a significant negative impact on the health of the elderly. Additionally, household debt not only deteriorates physical health, but also leads to an increase in mental stress. Our findings are still robust after replacing the explanatory variables. The endogeneity problem is solved by the IV-Oprobit model and PSM model.
2. Elderly women are more likely to experience a higher impact of household debt than men. With a higher education level, older adults experience more mental stress as a result of household debt. However, only the physical health of older adults with a low education level is affected by household debt.
3. As household income increases, the impact of household debt on older adults rises and then falls, forming an inverted U-shape relationship. This indicates that older adults from middle-income households are the most affected by household debt.
4. The mechanism of interaction between household debt and the health level of the elderly was also explored. As household debt increases, older adults have to work longer hours or rejoin the labor market to maintain their daily household expenditure. Overworking or psychological stress eventually deteriorates older adults’ physical and mental health. At the same time, higher household debt also tightens households’ budgets, and lower medical expenditures deteriorate the health level of the elderly.

Population aging has gradually changed the population structure in China, and increasing household debt makes the elderly a more vulnerable group than younger people. In order to improve the living standards of the elderly, our findings have some policy implications:

1. The regulation of the real estate market should be restricted, and the income and wealth of the elderly stabilized. Because house prices are highly related to household debt in China, financial and real estate market policies should complement regional house prices and cut off the self-reinforcing mechanism in which house prices and debt push each other up.
2. The financial stress of middle-income households should be alleviated through transfer payments or policy subsidies. Through the heterogeneity analysis in this study, it can be seen that older people in middle-income households are the most affected by household debt. In this case, the government should effectively identify households with different income levels, and provide subsidies or tax relief for middle-income households, in order to increase the real income of such households.
3. The social security and medical insurance systems should be improved to protect the health of the elderly. Through the study of the mechanisms of influence, we found that labor participation and medical expenditure shrinkage are both important factors that affect the physical and mental health of the elderly. Therefore, the government should improve the social security system and pension expenditures to prevent elderly adults from returning to work. Medical insurance coverage should also be improved so that more health problems can be covered by insurance.

## Figures and Tables

**Table 1:** Variable definitions.

Variables	Definition
Health	Self-rated health, which improves from 1 to 5
Depression	Depression score, which deteriorates from 6 to 24
Ln Debt	Total household debt
Age	Age in years
Gender	Male = 1; Female = 0
Education	Education in years
Urban	Urban = 1; Rural = 0
Drinking	Drinking = 1; No drinking = 0
Ln Income	Total household income
Ln Asset	Total household assets
Household Size	Number of family members

**Table 2:** Descriptive statistics.

Variables	Observations	Mean	Std. Dev	Min	Max
Health	7875	3.6889	1.2324	1	5
Depression	7875	9.8987	3.5291	6	24
Ln Debt	7875	2.4663	4.6384	0	15.4249
Age	7875	68.2160	6.3340	60	96
Gender	7875	0.5029	0.5000	0	1
Education	7875	4.5363	4.6281	0	19
Urban	7875	0.4702	0.5002	0	1
Drinking	7875	0.1751	0.3801	0	1
Ln Income	7875	10.2647	1.5649	0	15.4250
Ln Asset	7875	12.5613	1.5294	0	17.7367
Household Size	7875	3.8031	2.1390	1	21

**Table 3:** Oprobit regression analysis of the impact of household debt.

Variables	Health				Depression			
	(1)	(2)	(3)	(4)	(5)	(6)	(7)	(8)
	Oprobit	Oprobit	Oprobit	Oprobit	Oprobit	Oprobit	Oprobit	Oprobit
Ln Debt	−0.0044 * (0.0026)	−0.0055 ** (0.0026)	−0.0072 *** (0.0015)	−0.0043 *** (0.0010)	0.0155 *** (0.0025)	0.0149 *** (0.0025)	0.0210 *** (0.0028)	0.0234 *** (0.0030)
Age		−0.0087 *** (0.0019)	−0.0083 *** (0.0021)	−0.0084 *** (0.0021)		0.0052 *** (0.0019)	0.0042 ** (0.0020)	0.0039 ** (0.0017)
Gender		0.1679 *** (0.0270)	0.1670 *** (0.0285)	0.1649 *** (0.0288)		−0.2878 *** (0.0259)	−0.3194 *** (0.0273)	−0.3223 *** (0.0276)
Education		0.0065 ** (0.0029)	0.0044 (0.0031)	0.0057 (0.0048)		−0.0309 *** (0.0028)	−0.0236 *** (0.0030)	−0.0239 *** (0.0032)
Urban		0.0562 ** (0.0251)	0.0059 (0.0289)	0.0076 (0.0292)		−0.2437 *** (0.0240)	−0.1175 *** (0.0275)	−0.1197 *** (0.0278)
Drinking		−0.2529 *** (0.0334)	−0.2475 *** (0.0349)	−0.2515 *** (0.0353)		−0.1797 *** (0.0189)	−0.1663 *** (0.0339)	−0.1701 *** (0.0344)
Ln Income			0.0218 ** (0.0099)	0.0225 ** (0.0102)			−0.0773 *** (0.0094)	−0.0755 *** (0.0090)
Ln Asset			0.315 *** (0.0103)	0.0324 *** (0.0096)			−0.0787 *** (0.0099)	−0.0763 *** (0.0101)
Household Size			0.120 * (0.0067)	0.0131 ** (0.0064)			−0.0026 (0.0064)	−0.0038 (0.0065)
Province Fixed Effect	Control	Control	Control		Control	Control	Control	
Household Fixed Effect				Control				Control
Observations	7875	7875	7875	7875	7875	7875	7875	7875
Pseudo R^2^	0.0009	0.0097	0.0207	0.0216	0.0015	0.0182	0.0254	0.0252

Note: ***, **, and * represent significance at the 1%, 5%, and 10% level, respectively; robust standard errors are in parentheses.

**Table 4:** The marginal effects of household debt on the health of the elderly.

Health	Ln Debt	Depression	Ln Debt	Depression	Ln Debt
Health = 1	0.0024 ** (0.0010)	Depression = 6	−0.0052 *** (0.0007)	Depression = 16	0.0006 *** (0.0001)
Health = 2	0.0004 ** (0.0001)	Depression = 7	−0.0016 *** (0.0002)	Depression = 17	0.0006 *** (0.0001)
Health = 3	−0.0010 ** (0.0004)	Depression = 8	−0.0008 *** (0.0001)	Depression = 18	0.0005 *** (0.0001)
Health = 4	−0.0007 ** (0.0003)	Depression = 9	−0.0002 *** (0.00003)	Depression = 19	0.0004 *** (0.0001)
Health = 5	−0.0011 ** (0.0004)	Depression = 10	0.0005 *** (0.0001)	Depression = 20	0.0003 *** (0.0001)
		Depression = 11	0.0008 *** (0.0001)	Depression = 21	0.0001 *** (0.00004)
		Depression = 12	0.0011 *** (0.0001)	Depression = 22	0.0001 *** (0.00003)
		Depression = 13	0.0009 *** (0.0001)	Depression = 23	0.0001 *** (0.00003)
		Depression = 14	0.0008 *** (0.0001)	Depression = 24	0.0001 *** (0.00003)
		Depression = 15	0.0009 *** (0.0001)		

Note: *** and ** represent significance at the 1% and 5% level, respectively; robust standard errors are in parentheses.

**Table 5:** Empirical estimation results of the IV-Oprobit model.

Variables	(1)	(2)	(3)
	First-Stage	IV-Oprobit	IV-Oprobit
	Ln Debt	Health	Depression
Ln Debt		−0.0235 *** (0.0046)	0.0201 *** (0.0024)
Ln Debt16	0.8977 *** (0.0090)		
Control variables	Control	Control	Control
Province fixed effect	Control	Control	Control
Observations	7875	7875	7875
First-stage F-value	1160.89		
Prob > F	0		
Lnsig_2		1.0302 *** (0.0084)	1.4595 *** (0.0084)
Atanhrho_12		0.1716 *** (0.0134)	0.1097 *** (0.0153)

Note: *** represents significance at the 1% level; robust standard errors are in parentheses.

**Table 6:** Empirical results of propensity score matching.

	Matching Method	Treated	Controls	ATT	Standard Error	T-Value
Health	K-nearest neighbor	2.4688	2.5374	−0.0686	0.0127	−5.39 ***
	Caliper	2.4723	2.5633	−0.0910	0.0187	−4.87 ***
	Kernel	2.4697	2.5532	−0.0835	0.0182	−4.59 ***
Depression	K-nearest neighbor	10.3702	9.6395	0.7306	0.1201	6.09 ***
	Caliper	10.3745	9.6463	0.7282	0.1110	6.56 ***
	Kernel	10.3697	9.6526	0.7171	0.1094	6.55 ***

Note: *** represents significance at the 1% level.

**Table 7:** Empirical results of the robustness test.

Variables	(1)	(2)	(3)	(4)
	Oprobit	Oprobit	OLS	OLS
	Health	Depression	Health	Depression
Ln Household Debt	−0.0057 *** (0.0012)	0.0172 *** (0.0034)	−0.0038 *** (0.0009)	0.0102 *** (0.0028)
Control Variables	Control	Control	Control	Control
Province Fixed Effect	Control	Control	Control	Control
Observations	4725	4725	4725	4725

Note: *** represents significance at the 1% level; robust standard errors are in parentheses.

**Table 8:** Heterogeneity analysis results (gender differences).

Variables	(1)	(2)	(3)	(4)
	Female	Male	Female	Male
	Oprobit	Oprobit	Oprobit	Oprobit
	Health	Health	Depression	Depression
Ln Debt	−0.0149 *** (0.0043)	0.0003 (0.0040)	0.0216 *** (0.0039)	0.0205 *** (0.0039)
Control Variables	Control	Control	Control	Control
Province Fixed Effect	Control	Control	Control	Control
Observations	3886	3989	3886	3989

Note: *** represents significance at the 1% level; robust standard errors are in parentheses.

**Table 9:** Heterogeneity analysis results (education differences).

Variables	(1)	(2)	(3)	(4)	(5)	(6)
	Low Level	Medium Level	High Level	Low Level	Medium Level	High Level
	Oprobit	Oprobit	Oprobit	Oprobit	Oprobit	Oprobit
	Health	Health	Health	Depression	Depression	Depression
Ln Debt	−0.0085 ** (0.0033)	−0.0070 (0.0046)	0.0014 (0.0083)	0.0171 *** (0.0040)	0.0215 *** (0.0045)	0.0312 *** (0.0081)
Control Variables	Control	Control	Control	Control	Control	Control
Province Fixed Effect	Control	Control	Control	Control	Control	Control
Observations	3521	3213	1141	3521	3213	1141

Note: *** and ** represent significance at the 1% and 5% level, respectively; robust standard errors are in parentheses.

**Table 10:** Heterogeneity analysis results (income differences).

Variables	(1)	(2)	(3)	(4)	(5)	(6)
	Low Income	Middle Income	High Income	Low Income	Middle Income	High Income
	Oprobit	Oprobit	Oprobit	Oprobit	Oprobit	Oprobit
	Health	Health	Health	Depression	Depression	Depression
Ln Debt	−0.0061 *** (0.0021)	−0.0129 *** (0.0012)	−0.0047 ** (0.0011)	0.0100 * (0.0057)	0.0243 *** (0.0039)	0.0231 *** (0.0050)
Control Variables	Control	Control	Control	Control	Control	Control
Province Fixed Effect	Control	Control	Control	Control	Control	Control
Observations	1692	3781	1686	1692	3781	1686

Note: ***, **, and * represent significance at the 1%, 5%, and 10% level, respectively; robust standard errors are in parentheses.

**Table 11:** Household debt, labor, and health of the elderly.

Variables	(1)	(2)	(3)
	Probit	Oprobit	Oprobit
	Labor	Health	Depression
Labor		−0.3017 *** (0.0285)	0.1236 *** (0.0271)
Ln Debt	0.0241 *** (0.0028)	−0.0070 ** (0.0029)	0.0206 *** (0.0028)
Control variables	Control	Control	Control
Province fixed effect	Control	Control	Control
Observations	7875	7875	7875

Note: *** and ** represent significance at the 1% and 5% level, respectively; robust standard errors are in parentheses.

**Table 12:** Household debt, medical spending, and health of the elderly.

Variables	(1)	(2)	(3)
	OLS	Oprobit	Oprobit
	Ln Medical Expenditure	Health	Depression
Ln Medical Expenditure		0.1214 *** (0.0038)	−0.0627 *** (0.0035)
Ln Debt	−0.0046 ** (0.0023)	−0.0063 ** (0.0030)	0.0203 *** (0.0028)
Control Variables	Control	Control	Control
Province Fixed Effect	Control	Control	Control
Observations	7875	7875	7875

Note: *** and ** represent significance at the 1% and 5% level, respectively; robust standard errors are in parentheses.

## Data Availability

We used 2018 China Family Panel Studies (CFPS) data, which can be found at https://www.isss.pku.edu.cn/cfps/ (accessed on 22 September 2022).

## Appendix A

**Table A1:** Marginal effects of household debt in female subgroup.

Health	Ln Debt	Depression	Ln Debt	Depression	Ln Debt
Health = 1	0.0055 *** (0.0016)	Depression = 6	−0.0051 *** (0.0008)	Depression = 16	0.0008 *** (0.0002)
Health = 2	0.0004 *** (0.0001)	Depression = 7	−0.0019 *** (0.0004)	Depression = 17	0.0008 *** (0.0002)
Health = 3	−0.0023 *** (0.0007)	Depression = 8	−0.0012 *** (0.0002)	Depression = 18	0.0007 *** (0.0001)
Health = 4	−0.0014 *** (0.0004)	Depression = 9	−0.0004 *** (0.0001)	Depression = 19	0.0005 *** (0.0001)
Health = 5	−0.0022 *** (0.0006)	Depression = 10	0.0001 *** (0.00002)	Depression = 20	0.0004 *** (0.0001)
		Depression = 11	0.0006 *** (0.0001)	Depression = 21	0.0002 *** (0.0001)
		Depression = 12	0.0010 *** (0.0002)	Depression = 22	0.0001 *** (0.00004)
		Depression = 13	0.0012 *** (0.0002)	Depression = 23	0.0002 *** (0.00006)
		Depression = 14	0.0009 *** (0.0002)	Depression = 24	0.0002 *** (0.00007)
		Depression = 15	0.0009 *** (0.0002)		

Note: *** represents significance at the 1% level; robust standard errors are in parentheses.

**Table A2:** Marginal effects of household debt in male subgroup.

Health	Ln Debt	Depression	Ln Debt	Depression	Ln Debt
Health = 1	−0.0001 (0.0012)	Depression = 6	−0.0047 *** (0.0011)	Depression = 16	0.0004 *** (0.0001)
Health = 2	0 (0.0003)	Depression = 7	−0.0014 *** (0.0003)	Depression = 17	0.0003 *** (0.0001)
Health = 3	0 (0.0005)	Depression = 8	−0.0004 *** (0.0001)	Depression = 18	0.0003 *** (0.0001)
Health = 4	0 (0.0004)	Depression = 9	0.0003 *** (0.0001)	Depression = 19	0.0003 *** (0.0001)
Health = 5	0.0001 (0.0006)	Depression = 10	0.0008 *** (0.0001)	Depression = 20	0.0002 *** (0.0001)
		Depression = 11	0.0008 *** (0.0002)	Depression = 21	0.0001 *** (0.00004)
		Depression = 12	0.0010 *** (0.0002)	Depression = 22	0.0001 *** (0.00003)
		Depression = 13	0.0009 *** (0.0002)	Depression = 23	0.00005 * (0.00003)
		Depression = 14	0.0006 *** (0.0001)	Depression = 24	0.00001 (0.00001)
		Depression = 15	0.0005 *** (0.0001)		

Note: *** and * represent significance at the 1% and 10% level, respectively; robust standard errors are in parentheses.

**Table A3:** Marginal effects of household debt in low education subgroup.

Health	Ln Debt	Depression	Ln Debt	Depression	Ln Debt
Health = 1	0.0032 ** (0.0016)	Depression = 6	−0.0035 *** (0.0008)	Depression = 16	0.0006 *** (0.0002)
Health = 2	0.0002 * (0.0001)	Depression = 7	−0.0013 *** (0.0003)	Depression = 17	0.0006 *** (0.0002)
Health = 3	−0.0011 ** (0.0005)	Depression = 8	−0.0010 *** (0.0002)	Depression = 18	0.0006 *** (0.0002)
Health = 4	−0.0008 ** (0.0004)	Depression = 9	−0.0005 *** (0.0001)	Depression = 19	0.0005 *** (0.0001)
Health = 5	−0.0015 ** (0.0007)	Depression = 10	0.00001 (0.00003)	Depression = 20	0.0004 *** (0.0001)
		Depression = 11	0.0005 *** (0.0001)	Depression = 21	0.0002 *** (0.00007)
		Depression = 12	0.0007 *** (0.0002)	Depression = 22	0.0001 *** (0.00003)
		Depression = 13	0.0008 *** (0.0002)	Depression = 23	0.0001 ** (0.00004)
		Depression = 14	0.0007 *** (0.0002)	Depression = 24	0.0002 *** (0.00006)
		Depression = 15	0.0007 *** (0.0002)		

Note: ***, ** and * represent significance at the 1%, 5% and 10% level, respectively; robust standard errors are in parentheses.

**Table A4:** Marginal effects of household debt in medium education subgroup.

Health	Ln Debt	Depression	Ln Debt	Depression	Ln Debt
Health = 1	0.0024 (0.0022)	Depression = 6	−0.0061 *** (0.0016)	Depression = 16	0.0007 *** (0.0002)
Health = 2	0.0005 (0.0006)	Depression = 7	−0.0018 *** (0.0005)	Depression = 17	0.0006 *** (0.0002)
Health = 3	−0.0011 (0.0009)	Depression = 8	−0.007 *** (0.0002)	Depression = 18	0.0005 *** (0.0002)
Health = 4	−0.0008 (0.0006)	Depression = 9	0.0001 (0.0001)	Depression = 19	0.0002 ** (0.0001)
Health = 5	−0.0011 (0.0009)	Depression = 10	0.0008 *** (0.0002)	Depression = 20	0.0002 ** (0.0001)
		Depression = 11	0.0011 *** (0.0003)	Depression = 21	0.0002 *** (0.00007)
		Depression = 12	0.0012 *** (0.0003)	Depression = 22	0.00007 (0.00004)
		Depression = 13	0.0011 *** (0.0003)	Depression = 23	0.0001 (0.00007)
		Depression = 14	0.0008 *** (0.0002)	Depression = 24	0.00014 * (0.00008)
		Depression = 15	0.0008 *** (0.0002)		

Note: ***, ** and * represent significance at the 1%, 5% and 10% level, respectively; robust standard errors are in parentheses.

**Table A5:** Marginal effects of household debt in high education subgroup.

Health	Ln Debt	Depression	Ln Debt	Depression	Ln Debt
Health = 1	0.0008 (0.0015)	Depression = 6	−0.0078 *** (0.0015)	Depression = 16	0.0008 *** (0.0002)
Health = 2	0.0002 (0.0005)	Depression = 7	−0.0017 *** (0.0003)	Depression = 17	0.0005 *** (0.0001)
Health = 3	−0.004 (0.0008)	Depression = 8	−0.0003 *** (0.0001)	Depression = 18	0.0004 *** (0.0001)
Health = 4	−0.0003 (0.0006)	Depression = 9	−0.00005 (0.00004)	Depression = 19	0.0003 *** (0.0001)
Health = 5	−0.0004 (0.0007)	Depression = 10	0.0006 *** (0.0001)	Depression = 20	0.0001 ** (0.00007)
		Depression = 11	0.0014 *** (0.0003)	Depression = 21	0.00009 * (0.00005)
		Depression = 12	0.0015 *** (0.0003)	Depression = 22	0.00003 (0.00003)
		Depression = 13	0.0019 *** (0.0004)	Depression = 23	0.00009 * (0.00006)
		Depression = 14	0.0010 *** (0.0002)	Depression = 24	0.0001 * (0.00006)
		Depression = 15	0.0011 *** (0.0003)		

Note: ***, ** and * represent significance at the 1%, 5% and 10% level, respectively; robust standard errors are in parentheses.

**Table A6:** Marginal effects of household debt in low income subgroup.

Health	Ln Debt	Depression	Ln Debt	Depression	Ln Debt
Health = 1	0.0021 *** (0.0007)	Depression = 6	−0.0020 ** (0.00011)	Depression = 16	0.0004 ** (0.0002)
Health = 2	0.0003 ** (0.0002)	Depression = 7	−0.0007 * (0.0005)	Depression = 17	0.0004 * (0.0003)
Health = 3	−0.0010 ** (0.0006)	Depression = 8	−0.0006 * (0.0004)	Depression = 18	0.0004 ** (0.0002)
Health = 4	−0.0008 ** (0.0005)	Depression = 9	−0.0004 * (0.0003)	Depression = 19	0.0003 * (0.0002)
Health = 5	−0.0006 ** (0.0004)	Depression = 10	−0.0001 * (0.00007)	Depression = 20	0.0003 * (0.0002)
		Depression = 11	0.0001 * (0.00006)	Depression = 21	0.0002 ** (0.0001)
		Depression = 12	0.0004 * (0.0003)	Depression = 22	0.00008 * (0.00005)
		Depression = 13	0.0003 ** (0.0002)	Depression = 23	0.0001 * (0.00007)
		Depression = 14	0.0003 *** (0.0001)	Depression = 24	0.0001 * (0.00006)
		Depression = 15	0.0004 *** (0.0001)		

Note: ***, ** and * represent significance at the 1%, 5% and 10% level, respectively; robust standard errors are in parentheses.

**Table A7:** Marginal effects of household debt in middle income subgroup.

Health	Ln Debt	Depression	Ln Debt	Depression	Ln Debt
Health = 1	0.0049 *** (0.0017)	Depression = 6	−0.0069 *** (0.0015)	Depression = 16	0.0006 *** (0.0002)
Health = 2	0.0002 *** (0.0001)	Depression = 7	−0.0017 *** (0.0004)	Depression = 17	0.0005 *** (0.0002)
Health = 3	−0.0018 *** (0.0004)	Depression = 8	−0.0002 ** (0.0001)	Depression = 18	0.0003 *** (0.0001)
Health = 4	−0.0012 *** (0.0003)	Depression = 9	−0.0001 *** (0.00002)	Depression = 19	0.0002 *** (0.00004)
Health = 5	−0.0020 *** (0.0005)	Depression = 10	0.0007 *** (0.0002)	Depression = 20	0.0002 *** (0.00005)
		Depression = 11	0.0014 *** (0.0003)	Depression = 21	0.0002 *** (0.00006)
		Depression = 12	0.0014 *** (0.0003)	Depression = 22	0.0001 *** (0.00003)
		Depression = 13	0.0014 *** (0.0003)	Depression = 23	0.0001 *** (0.00002)
		Depression = 14	0.0011 *** (0.0002)	Depression = 24	0.0001 *** (0.00002)
		Depression = 15	0.0008 *** (0.0002)		

Note: *** and ** represent significance at the 1% and 5% level, respectively; robust standard errors are in parentheses.

**Table A8:** Marginal effects of household debt in high income subgroup.

Health	Ln Debt	Depression	Ln Debt	Depression	Ln Debt
Health = 1	0.0014 *** (0.0003)	Depression = 6	−0.0061 *** (0.0010)	Depression = 16	0.0007 *** (0.0001)
Health = 2	0.0003 *** (0.0001)	Depression = 7	−0.0019 *** (0.0003)	Depression = 17	0.0008 *** (0.0001)
Health = 3	−0.0006 *** (0.0002)	Depression = 8	−0.0010 *** (0.0002)	Depression = 18	0.0006 *** (0.0001)
Health = 4	−0.0006 *** (0.0001)	Depression = 9	−0.0002 *** (0.00006)	Depression = 19	0.0004 *** (0.0001)
Health = 5	−0.0005 *** (0.0001)	Depression = 10	0.0006 *** (0.0001)	Depression = 20	0.0003 *** (0.0001)
		Depression = 11	0.0010 *** (0.0002)	Depression = 21	0.0002 *** (0.0001)
		Depression = 12	0.0013 *** (0.0002)	Depression = 22	0.0001 *** (0.00002)
		Depression = 13	0.0012 *** (0.0002)	Depression = 23	0.0001 *** (0.00002)
		Depression = 14	0.0011 *** (0.0002)	Depression = 24	0.0001 *** (0.00003)
		Depression = 15	0.0009 *** (0.0002)		

Note: *** represents significance at the 1% level; robust standard errors are in parentheses.
